# High nitrogen solubility in stishovite (SiO_2_) under lower mantle conditions

**DOI:** 10.1038/s41598-020-67621-2

**Published:** 2020-07-02

**Authors:** Ko Fukuyama, Hiroyuki Kagi, Toru Inoue, Sho Kakizawa, Toru Shinmei, Shunichi Hishita, Naoto Takahata, Yuji Sano

**Affiliations:** 1 Geochemical Research Center, Graduate School of Science, The University of Tokyo, Hongo, Tokyo, 113-0033 Japan; 2 Department of Earth and Planetary Systems Science, Hiroshima University, Higashi-Hiroshima, Hiroshima 739-8526 Japan; 3 Geodynamics Research Center, Ehime University, Matsuyama, Ehime 790-8577 Japan; 4 Research and Services Division of Materials Data and Integrated System, National Institute for Materials Science, Tsukuba, Ibaraki 305-0047 Japan; 5 Atmosphere and Ocean Research Institute, The University of Tokyo, Kashiwa, Chiba 277-8564 Japan; 6 Institute of Surface-Earth System Science, Tianjin University, Tianjin, 300072 People’s Republic of China

**Keywords:** Geochemistry, Mineralogy

## Abstract

Nitrogen is a crucial volatile element in the early Earth’s evolution and the origin of life. Despite its importance, nitrogen’s behavior in the Earth's interior remains poorly understood. Compared to other volatile elements, nitrogen is depleted in the Earth’s atmosphere (the so-called “missing nitrogen”), calling for a hidden deep reservoir. To investigate nitrogen’s behavior in the deep Earth including how the reservoir formed, high-pressure and high-temperature experiments were conducted at 28 GPa and 1,400–1,700 °C. To reproduce the conditions in the lower mantle, the redox was controlled using a Fe–FeO buffer. We observed that depending on the temperature conditions, stishovite can incorporate up to 90–404 ppm nitrogen, experimentally demonstrating that stishovite has the highest nitrogen solubility among the deep mantle minerals. Stishovite is the main mineral component of subducted nitrogen-rich sedimentary rocks and eroded continental crust that are eventually transported down to the lower mantle. Our results suggest that nitrogen could have been continuously transported into the lower mantle via subduction, ever since plate tectonics began.

## Introduction

Molecular nitrogen (N_2_) constitutes approximately 78% of the Earth’s present atmosphere and is an essential element for sustaining life. It plays a vital role in the N biogeochemical cycle because molecular nitrogen (N_2_) is fixed by microorganisms as ammonium (NH_4_^+^) and successively transformed into nitrates (NO_3_^−^) in the Earth superficial reservoirs^1^. Past variations in the atmospheric content of nitrogen, which is still under debate, may have played an essential role in regulating the early Earth’s climate^2,3^ as greenhouse gas. Uncertainties in understanding the secular changes in the Earth’s atmospheric nitrogen concentration are partially caused by the lack of knowledge about the deep geological cycle of nitrogen and particularly its abundance and chemical form in the terrestrial mantle^4^. Compared to other volatile elements, nitrogen is depleted by one order of magnitude in the mantle and in the whole Earth^5^. This is the so-called “missing nitrogen”. However, the causes of the nitrogen depletion, compared to the terrestrial volatile inventory, have not been yet identified. One hypothesis suggests losses from the early Earth’s atmosphere by meteorite impacts^6^. However, meteorites and comets have likely delivered nitrogen to the proto-Earth^7,8^, making difficult to explain the “missing nitrogen” solely by atmospheric dissipation. A second hypothesis proposes the existence of a nitrogen reservoir in the deep Earth. For example, nitrides such as cubic boron nitride, titanium nitride, and iron nitride have been discovered respectively as inclusions of ophiolite^9,10^ or of super-deep diamonds originating from the upper mantle and the lower mantle^11,12^. Previous studies suggested that there are possible avenues for nitrogen reservoir formation in the deep Earth. Magma ocean^6,13–17^ and subduction are an event and a process which formed nitrogen reservoir in the deep Earth^4,18–21^.

During the magma ocean, nitrogen was thought to dissolve into liquid iron because of its moderately siderophile behavior^14,22,23^. However, Dalou et al.^6^ reported that a high carbon to nitrogen ratio (C/N = 6.1 × 10^2^ from Marty^5^) in the bulk silicate Earth (BSE) cannot be solely explained by the core formation because carbon is more siderophile than nitrogen under an oxygen fugacity (fO_2_) of IW-0.4 to IW-3.5 (IW: Fe–FeO buffer). If a major nitrogen reservoir was formed in the Earth’s core, then nitrogen would not be depleted relative to carbon in BSE, which conflicts with the nitrogen depletion compared to carbon in the BSE, as reported by Marty^5^. First principle molecular dynamic simulations clarified that nitrogen solely cannot explain both the density and the bulk sound velocity of the outer core^24^. However, there is a possibility that the Earth’s core plays an important role in reserving nitrogen. Li et al.^13^ conducted high-pressure experiments under Ni–NiO and Fe–FeO buffers, reporting that the deeper part of the upper mantle can become a nitrogen reservoir through the solidification of a magma ocean because the maximum nitrogen solubilities in forsterite and enstatite were determined as ca. 10 ppm and 100 ppm^13^, respectively. In addition, Yoshioka et al*.*^15^ reported a 21.5 ± 18.1 ppm nitrogen solubility in bridgmanite, in high-pressure experiments with a multi-anvil apparatus and under reduced conditions close to the Fe–FeO buffer. This is the only study of the nitrogen solubility of bridgmanite which, occupies approximately 80 vol% of the lower mantle^25^.

A nitrogen reservoir might have also been formed in the mantle by subducting slabs^26^, because the inward nitrogen flux of subduction is one order of magnitude higher than that of the outgassing flux^27–30^. The Earth is the only planet in the Solar system with active plate tectonics^31^. Such volatile cycle via subduction has remained a very important research topic in geochemistry. To date, various NH_4_-bearing silicate minerals have been synthesized under conditions corresponding to cold subducting slabs in the deep upper mantle^4,18,20,21^. Sediments and eroded continental crust in subducting slabs can be nitrogen-rich. Average concentrations of nitrogen in sediments and upper crusts are, respectively, 560 ppm and 150 ppm^32^. However, experimental conditions reported in earlier studies^4,18,20,21^ are limited to the deep upper mantle (< 13 GPa). Consequently, nitrogen behavior in the mantle transition zone (> 13 GPa) and in the lower mantle (> 24 GPa) are still unknown. Therefore, we conducted high-pressure experiments at lower mantle conditions to investigate nitrogen incorporation into deep mantle minerals such as stishovite and bridgmanite.

## Results

### Description of the run products

**Figure 1:**
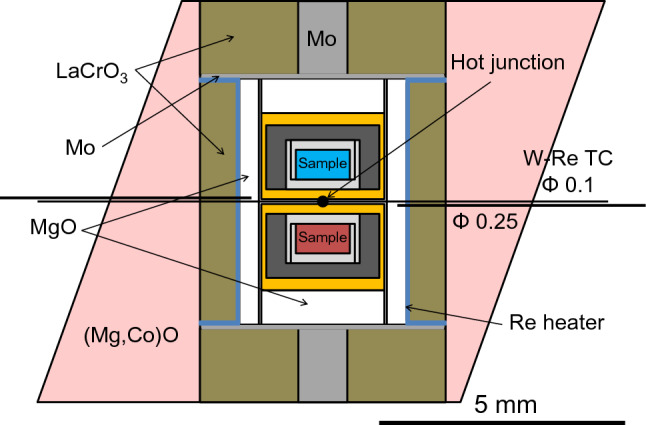
Cell assembly for high-pressure and high-temperature experiments.

**Figure 2:**
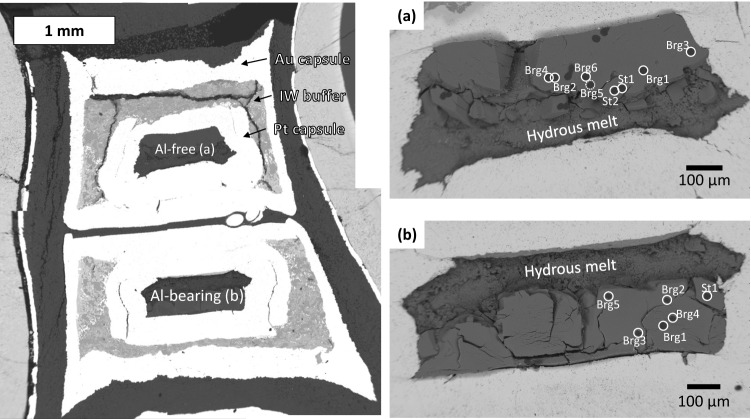
(Left) Back scattered electron (BSE) image of recovered sample from 28 GPa and 1,400 °C (OT2259). (Right) BSE images of two samples in OT2259: (**a**) Al-free system and (**b**) Al-bearing system. Circles indicate analyzed points. St denotes stishovite, Brg bridgmanite Brg2 and Brg4 in the Al-bearing sample contained iron. Dotted circles indicate analysis points located in cracks or intergranular, which are not discussed in this paper. This figure was created by Ko Fukuyama using Microsoft Power Point and Excel (OFFICE 2016).

The experimental conditions are presented in Table 1 and Supplementary Fig. S1. Figure 1 illustrates the cell assembly used for the high-pressure experiments. Figure 2 and Supplementary Figs. S3–S8 present the backscattered electron (BSE) images of recovered cell assemblies and recovered samples. Each outer gold (Au) capsule included an inner platinum (Pt) capsule surrounded by a Fe–FeO buffer. The two inner Pt capsules of the cell assembly accommodated the Al-free and Al-bearing samples, respectively. The outer Au and the inner Pt capsule partially melted during the runs, because of iron and water coexistence (Fig. 2 and Supplementary Figs. S3–S8). However, no Fe–FeO leaked out of the Au capsules. After all the runs were completed, a subsequent EDS analysis revealed that pure metallic iron was still present and FeO contained no Mg. The Fe and FeO coexistence after the runs indicated that the experiments’ oxygen fugacity conditions were that of the lower mantle. Therefore, we inferred that hydrogen was generated in the outer Au capsule and permeated the inner capsule. The MgO-rich hydrous melt in the inner Pt capsule was expected to contain ^15^NH_3_ under a hydrogen-coexisting environment. Run products consisted of stishovite, bridgmanite, and a hydrous melt. The resulting single crystals of stishovite and bridgmanite were larger than 40 μm and 80 μm, respectively. These grain sizes were sufficiently large to be analyzed by nanoscale secondary ion mass spectrometry (NanoSIMS). Some bridgmanite samples contained iron with concentrations up to approximately 1.75 at% in Al-bearing samples (1,500 °C, OT2258; 1,400 °C, OT2259; 1,620 °C, OT2293). Iron contamination from the Fe–FeO buffer might result from gaps between the Pt double capsules or the iron diffusion into the capsule. The quenched hydrous melt was found to be MgO-rich for all recovered samples (e.g. Si/Mg ratio = 0–0.25 in OS3083).

**Table 1:** Experimental conditions and run products in recovered samples.

Run no.	Pressure (GPa)	Temperature (°C)	Duration time (min)	Outer capsule	Inner capsule	Buffer	Run products
OS3083	28	1,700	120	Au	Pt	Fe–FeO	Brg, St, melt
OT2258	28	1,500	120	Au	Pt	Fe–FeO	Brg, St, melt
OT2259	28	1,400	120	Au	Pt	Fe–FeO	Brg, St, melt
OT2293	28	1,620	120	Au	Pt	Fe–FeO	Brg, St, melt

### Nitrogen in stishovite and bridgmanite and its solubility in stishovite under various temperatures

We compared the respective nitrogen contents of stishovite and bridgmanite recovered from the run products by looking at the ^15^N^16^O^−^/^30^Si^−^ ratio. Because of the insufficient mass resolution of the NanoSIMS (mass resolving power *M*/Δ*M* ≒ 4,000), the ^15^N^16^O^−^ and ^29^SiH_2_^−^ ions could not be separated. Therefore, we exclusively calculated the ^15^N^16^O^−^ ion counts by subtracting the potential ^29^SiH_2_^−^ ion counts. These latter have been estimated from the ^28^SiH_2_^−^ ion counts based on the ^29^Si^−^/^28^Si^−^ isotopic ratio (5.06 × 10^–2^). The Supplementary Figure S9 shows the time-dependence of the representative ion counts. In stishovite, the ^15^N^16^O^−^ ion counts were higher than ^28^SiH_2_^−^ ion counts, indicating that the contribution of the ^29^SiH_2_^−^ to ^15^N^16^O^−^ was negligible. Contrastingly, in bridgmanite, the ^15^N^16^O^−^ ion counts were much lower than those of ^28^SiH_2_^−^. The estimated ^29^SiH_2_^−^ ion counts were 65.1% of the original ^15^N^16^O^−^ ion counts at maximum (Supplementary Fig. S9b). Therefore, in bridgmanite, we were able to estimate only the maximum possible concentration.

**Figure 3:**
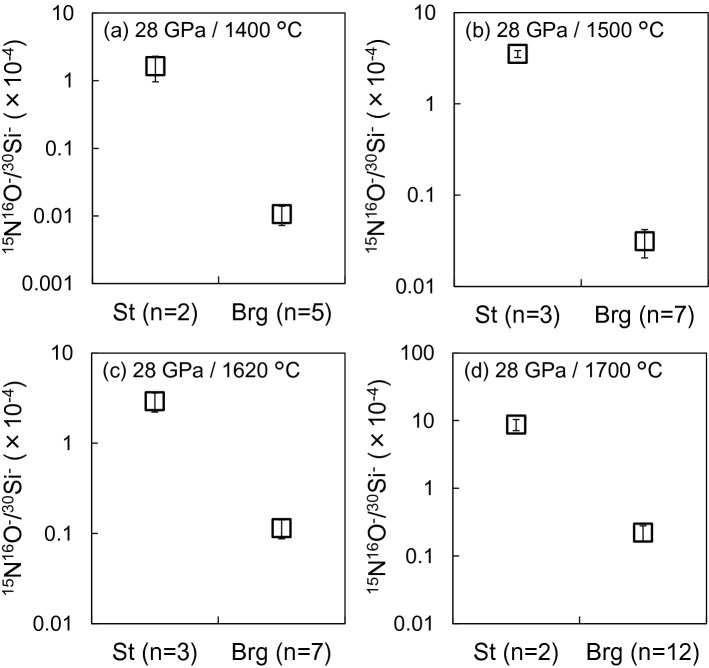
^15^N^16^O^−^ to ^30^Si^−^ count ratio in the Al-free system; samples recovered from (**a**) 28 GPa, 1,400 °C (OT2259); (**b**) 28 GPa, 1,500 °C (OT2258); (**c**) 28 GPa, 1,620 °C (OT2293); (**d**) 28 GPa, 1,700 °C (OS3083). St denotes stishovite and Brg, bridgmanite; *n* is the number of analyzed points; error bars represent the standard deviation. This figure was created by Ko Fukuyama using Microsoft Power Point and Excel (OFFICE 2016).

Figure 3 and Supplementary Fig. S10 present nitrogen incorporation rates in stishovite and bridgmanite, respectively, in both the Al-free and Al-bearing systems. These results suggest that the nitrogen solubility of stishovite is markedly higher than that of bridgmanite, for both systems. Although nitrogen solubilities of different silicate minerals from ^15^N^16^O^−^/^30^Si^−^ cannot be compared directly because of a matrix effect, it is noteworthy that the ion intensity ratio of stishovite was one to two orders of magnitude larger than that of bridgmanite, in all recovered samples (Fig. 3 and Supplementary Fig. S10). The nitrogen solubility in stishovite in the Al-free system can be higher than that in the Al-bearing system (see Supplementary Fig. S11). Due to the low counts of ^15^N^16^O ions, the nitrogen solubility in bridgmanite between the Al-free and Al-bearing systems was difficult to evaluate.

**Figure 4:**
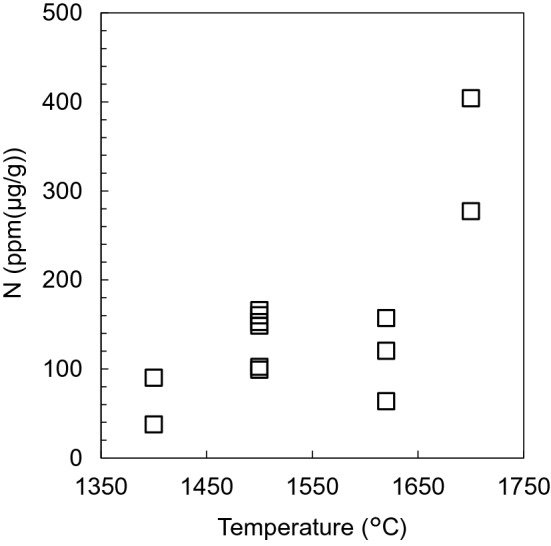
Nitrogen solubility in stishovite at different temperatures. All points refer to single measurement points of the sample; errors were obtained using the square root of ^15^N^16^O^−^ ion counts. This figure was created by Ko Fukuyama using Microsoft Power Point (OFFICE 2016).

Figure 4 presents the nitrogen solubility in stishovite, estimated using nitrogen-implanted quartz glass as standard samples. The nitrogen solubility in stishovite varied from 90 ppm (μg/g) to 404 ppm (μg/g). Stishovite can incorporate higher concentrations of nitrogen than bridgmanite (21.5 ± 18.1 ppm)^15^. In addition, the nitrogen solubility in stishovite increases with temperature. This implies that nitrogen might be incorporated in the stishovite crystal structure as N^3−^ into oxygen vacancies or as N_2_ into Schottky defects^13^ (lattice point defects formed by unoccupied cation and anion sites in a stoichiometric ratio). Although oxygen vacancies might be more numerous in crystal structures at higher temperatures, substitutions between N^3−^ and O^2−^ require charge compensation. In stishovite, nitrogen is not expected to exist as NH_4_^+^ because NH_4_-bearing silicate minerals release nitrogen at temperatures of 750–1,000 °C^20,33,34^ much lower than those of our experimental conditions. Nitrogen solubilities in mantle minerals were investigated from high-pressure and high-temperature experiments: e.g. forsterite (7.1 ± 1.6 ppm), Al-bearing enstatite (50–100 ppm), wadsleyite (188.8 ± 15.6 ppm), ringwoodite (110.4 ± 109.8 ppm), bridgmanite (21.5 ± 18.1 ppm), and Ca-perovskite (28.3 ± 23.6 ppm)^13,15^. Nitrogen solubility in stishovite can be higher than in these minerals.

## Discussion

Metamorphism of SiO_2_-rich sediments and nitrogen-containing continental crust form stishovite in subducting slabs. Moreover, Lee et al.^35^ reported that subduction erosion plays an important role in nitrogen transport into the mantle based on the nitrogen isotope data. The subduction erosion invokes the removal of continental material at the frontal or basal areas of continental margins. The mineral proportion of clinopyroxene, which can incorporate a significant quantity of nitrogen^4,13^ in subducted sediments and eroded continental crust, decreases with increasing depth^36^. In addition, NH_4_-bearing silicate minerals are unstable at high temperatures^20,33,34^. Therefore, stishovite, which can incorporate nitrogen at higher temperatures up to 1,700 °C, is the major mineral in the subducted sediment and, presumably, in the subducted eroded continental crust. Ingalls et al.^37^ reported that continental crusts can subduct into the mid upper mantle in spite of its high buoyancy. Moreover, stishovite has the highest nitrogen solubility in major minerals formed by the phase transition in subducting slabs. We propose stishovite as the major nitrogen carrier down to the transition zone and the lower mantle, if stishovite can inherit nitrogen from clinopyroxene^4,13^ or some NH_4_-bearing high-pressure minerals^18^ which are stable up to 10 GPa and 700 °C^18,36^. This P–T condition corresponds to a depth of 300 km where stishovite forms from metamorphism of the subducted sediments.

Based on the mass of subducted material, we estimated the mass of nitrogen exclusively transported by stishovite. Current subduction rates derived from sediments and continental crusts are, respectively, 1.65 km^3^/year and 2.1 km^3^/year, consisting of > 1.7 km^3^/year from subduction erosion and 0.4 km^3^/year from crustal subduction during continental collision^38^. Stishovite accounts for about 40 vol% of the subducted sediment and about 30 vol% of the continental crust, at pressures higher than 10 GPa or depths greater than 300 km^36^. Stishovite can incorporate up to 90 μg/g nitrogen at 28 GPa and 1,400 °C (see Fig. 4), corresponding to the present cold slab geotherm^39^. From these parameters, we estimated that stishovite can transport a maximum nitrogen quantity of 5.0 × 10^8^ kg/year into the mantle transition zone and the lower mantle. If stishovite can transport nitrogen into the lower mantle, then almost all the transported nitrogen can be retained there. This is because the current nitrogen outgassing from the lower mantle [OIB (Ocean island basalt): 1.15 × 10^5^ kg/year] is three orders of magnitude lower than the nitrogen amount transported into the lower mantle by stishovite^28^. Thus, the maximum estimation of nitrogen transported into the lower mantle ranged from 0.46 to 0.53 PAN (PAN: Present Atmospheric Nitrogen) since 3.6–4.2 billion years ago, when plate tectonics started^40–43^ although these start times of plate tectonics are controversial. Furthermore, outgassing C/N ratios (mol) from subduction (C/N = 5.2 × 10^3^) and OIB (C/N = 7.1 × 10^3^) are approximately one order of magnitude higher than that from MORB (Mid-ocean ridge basalt, C/N = 6.8 × 10^2^)^28,44^. Bridgmanite and metallic iron in the lower mantle incorporate nitrogen^15,23^ and can store a maximum of 25 PAN^15^; this nitrogen storage capacity is greater than that of either the upper mantle or the transition zone^15^. Subduction is expected to play an essential role in the co-evolution between the atmosphere and the deep mantle, especially in the lower mantle. While metallic iron in the reduced lower mantle is expected to play a crucial role in reserving much nitrogen^23^, silicate minerals are the only nitrogen reservoir in subduction zones under oxidized conditions. K-hollandite and garnet are formed by the transition of the subducted sediments and continental crust in the mantle. These minerals can also incorporate nitrogen, as Watenphul et al.^18^ and Li et al.^13^ reported based on the experiments carried under lower pressures corresponding to those of the upper mantle. Although nitrogen solubilities in K-hollandite and garnet have not been estimated under the high-pressure corresponding to the lower mantle, the maximum of the nitrogen mass transported into the lower mantle via subducting slabs might be greater than the estimates solely based on stishovite transport. Compared to carbon, the formation of nitrogen reservoir in the lower mantle can deplete nitrogen effectively and might explain the “missing” nitrogen.

**Figure 5:**
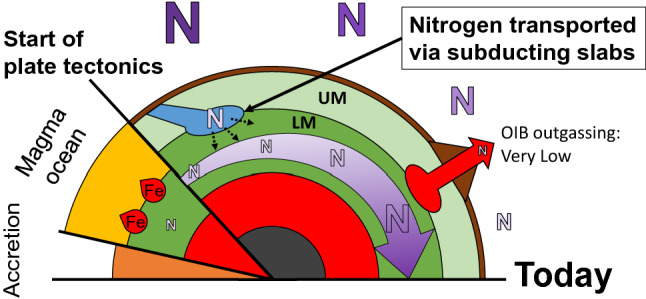
Deep “hidden” nitrogen reservoir formed by subducting slabs. *UM* the upper mantle, *LM* lower mantle, *OIB* oceanic island basalt.

Nitrogen concentration in the Archean atmosphere remains controversial. Some researchers have reported that the partial pressure of nitrogen (pN_2_) was half as low as that of the present atmosphere^45–47^. Others suggested that the partial pressure of nitrogen (pN_2_) in the Archean atmosphere might have been 1.4–3 times higher than that at present. The whole Earth was not frozen in the Archean Earth despite the fainter Sun^48^. The higher pN_2_ in the atmosphere might reconcile the faint young Sun paradox^2^: greater nitrogen contents in the early Earth’s atmosphere might have enhanced the greenhouse effect of CO_2_^2^. Goldblatt et al.^2^ suggested that nitrogen was transported into the deep Earth via subduction zones lowering the higher initial concentration in the atmosphere down to the present level. These results are supported by high-pressure experiments of Mallik et al. reporting that the Archean atmosphere had a 1.4–1.6 times^19^. Barry and Hilton^49^ also reported that the early atmospheric N content was approximately 50% higher than it is today based on a model using N–He–Ne–Ar isotope data Our results are consistent with the previous studies^2,19,49^ reporting that the mass of nitrogen in the ancient atmosphere was greater than that at present if the fluxes of nitrogen outgassing from the ancient and the present OIB were similar. Nitrogen transport into the lower mantle by stishovite and the formation of a deep nitrogen reservoir might have affected the global nitrogen cycle and the history of the Earth’s atmosphere. If the lower mantle could capture the nitrogen transported by stishovite, then a deep “hidden” nitrogen reservoir has formed in the lower mantle since the plate tectonics started (Fig. 5). Stishovite might have played a key role in the co-evolution of the atmosphere and the deep mantle.

## Methods

### High-pressure experiments using a multi-anvil apparatus

High-pressure and high-temperature experiments were conducted at the Geodynamics Research Center, Ehime University, Japan. We used a Kawai-type 2000 tons multi-anvil apparatus (Orange-2000) and a Kawai-type 3,000 tons multi-anvil apparatus (Orange-3000) installed at the Geodynamics Research Center, Ehime University, Japan. All experiments were conducted at 28 GPa and 1,400–1,700 °C, for a 2 h heating duration.

We used tungsten carbide anvils (Tungaloy F-grade and Fujilloy F08) with 4 mm truncated edge length (TEL). Two kinds of starting materials were prepared: a powdered mixture of MgO and SiO_2_ (quartz) for the ideal bridgmanite composition (MgSiO_3_), and a powdered mixture of Al_2_O_3_, MgO, Mg(OH)_2_, and SiO_2_ (quartz) for the Al-bearing hydrous bridgmanite composition (MgSi_0.9_Al_0.1_H_0.1_O_3_). From the hydrous starting material, we intended to grow the large crystals of stishovite and bridgmanite necessary for SIMS measurements. To distinguish sample-derived nitrogen from atmospheric or instrumental contamination, more than 99.6% ^15^N-substituted ammonium nitrate (^15^NH_4_^15^NO_3_, SHOKO SCIENCE Corp.) was used as nitrogen source. Contamination with atmospheric ^15^N is negligible because the ^15^N natural abundance is more than two orders of magnitude lower than that of the atmospheric ^14^N. Starting materials and ^15^NH_4_^15^NO_3_ were enclosed in a Pt capsule. The starting material to nitrogen source mass ratio was approximately 5:1 for each experiment. For run no. OS3083, the starting material was separated from the ^15^NH_4_^15^NO_3_ by 30 μm thick Au foil. For the other runs, the starting material and ^15^NH_4_^15^NO_3_ were mixed. The Pt capsule was surrounded by a Fe–FeO buffer, which contained water to reproduce the oxygen fugacity corresponding to the lower mantle^50–52^ and enclosed in an Au outer capsule. The two Au capsules of the cell assembly were insulated from the Re heater by a 25 μm thick magnesia sleeve. We used 150 mesh iron powders and iron oxide (FeO) powders with particle sizes smaller than 200 mesh for the Fe–FeO buffer (Fe:FeO = 2:1, wt%); 20–50 μl of water were added per each 0.5 g Fe–FeO. As previously described, Fig. 1 depicts the cell assembly used for this study. Thermal insulation was provided by a LaCrO_3_ sleeve. The Pt sample capsule was produced combining two Pt tubes, with 0.1 mm thick walls and outer diameters of 1.3 and 1.5 mm, by welding each end of the capsules^53^. An Au capsule was made from an Au tube with 0.1 mm wall thickness and 2.5 mm outer diameter. The temperature was measured by a W–Re (W3%Re–W25%Re) thermocouple inserted in the octahedron, attached to the Au capsules. Hydrogen fugacity in the inner and outer capsules was assumed to be equal because Pt has a high hydrogen permeability compared to Au. At high temperatures, ^15^NH_4_^15^NO_3_ decomposes into ^15^N_2_O and H_2_O; under the fO_2_ condition, ^15^NH_3_ is expected to form in ^15^N–H–O fluid under the fO_2_ condition in the inner Pt capsule.

The chemical composition of the quenched minerals was analyzed using a FE-SEM (JSM-7000F; JEOL) under operating conditions of 15 kV and 87.4–130.4 μA. For phase evaluation, Raman spectra were obtained using a micro-Raman spectrometer with an Ar ion laser of 514.5 nm and 6 mW power. Both characterizations were performed at the University of Tokyo, Japan.

### Nitrogen analysis of silicate minerals using NanoSIMS

A quantitative nitrogen analysis was conducted using a NanoSIMS 50 (Cameca) installed at the Atmosphere and Ocean Research Institute, The University of Tokyo (AORI), Japan. This NanoSIMS has a magnetic sector mass analyzer with seven parallel detection systems to simultaneously detect up to seven ion species as secondary ions. We employed a Cs^+^ primary ion beam with a 2 nA current, 5 μm diameter, and a 5 μm × 5 μm or 10 μm × 10 μm raster. We detected nitrogen as ^15^N^16^O^−^, as described by Li et al.^11^ To avoid charge-up, an e^−^ gun was used at 500–800 nA. Count times for the detection of ^30^Si^−^, ^30^SiH^−^, ^28^SiH_2_^−^, and ^15^N^16^O^−^were, respectively, 2 s, 2 s, 10 s, and 10 s. The time of analysis varied between 600 and 1,500 s. The post-bombardment crater volume was measured using laser scanning microscopy (Olympus Corp.).

We prepared ion-implanted standard samples to estimate the nitrogen concentrations in stishovite at the National Institute for Materials Science (NIMS). We used ^14^N-implanted quartz glass as the nitrogen standard for stishovite. The irradiation of ^14^N^+^ ions into the quartz glass was performed at 132 keV, with doses of 2.44 × 10^14^ and 3.66 × 10^15^ ions/cm^2^ (Ion Implanter RD-200l; Nissin Electric Co. Ltd.). Supplementary Figure S2 presents the calibration line for the nitrogen analysis using NanoSIMS.

## Supplementary information

Supplementary information.
