# Supplementary material for: High nitrogen solubility in stishovite (SiO2) under lower mantle conditions
Source: Sci Rep. 2020 Jul 2;10:10897. doi: 10.1038/s41598-020-67621-2 (PMC7331719; doi:10.1038/s41598-020-67621-2)
Supplement: Supplementary file 1 — Supplementary information. [file 41598_2020_67621_MOESM1_ESM.docx]

**High nitrogen solubility in stishovite (SiO_2_)**

**under the lower mantle conditions**

Ko Fukuyama^* 1^, Hiroyuki Kagi^1^, Toru Inoue^2, 3^, Sho Kakizawa^1^, Toru Shinmei^3^, Shunichi Hishita^4^, Naoto Takahata^5^, Yuji Sano^5,6^

^1^ Geochemical Research Center, Graduate School of Science, The University of Tokyo, Hongo, Tokyo 113-0033, Japan

^2^ Department of Earth and Planetary Systems Science, Hiroshima University, Higashi-Hiroshima, Hiroshima 739-8526, Japan

^3^ Geodynamics Research Center, Ehime University, Matsuyama, Ehime 790-5877, Japan

^4^ Research and Services Division of Materials Data and Integrated System, National Institute for Materials Science, Tsukuba, Ibaraki 305-0047, Japan

^5^ Atmosphere and Ocean Research Institute, The University of Tokyo, Kashiwa, Chiba, 277-8564, Japan

^6^ Institute of Surface-Earth System Science, Tianjin University, Tianjin, 300072, P.R. China

(*[ko.fukuyama@eqchem.s.u-tokyo.ac.jp](mailto:ko.fukuyama@eqchem.s.u-tokyo.ac.jp))

**Supplementary Information**

Supplementary Figure: 10 figures

Supplementary References: 3 references


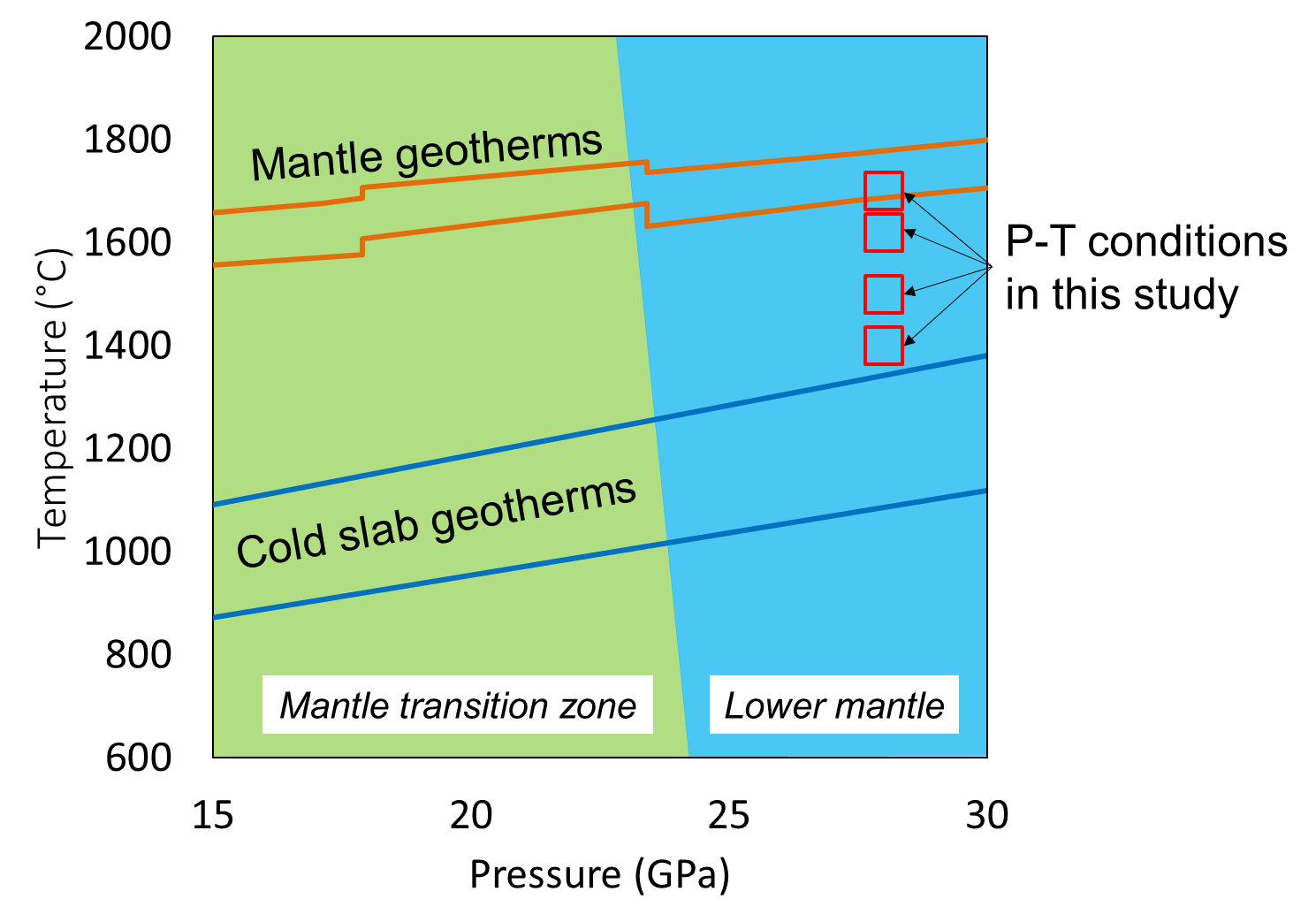


Supplementary Figure S1. Experimental *P-T* conditions in this study. Pressure condition in all experiments was 28 GPa and temperature conditions ranged from 1400 °C to 1700 °C. Mantle geotherms and slab geotherms in this figure were estimated by Katsura *et al*.^1^ and Liu *et al*.^2^ respectively. The slab geotherms were based on Syracuse *et al*.^3^.

This figure was created by Ko Fukuyama using Microsoft Power Point and Excel (OFFICE 2016).


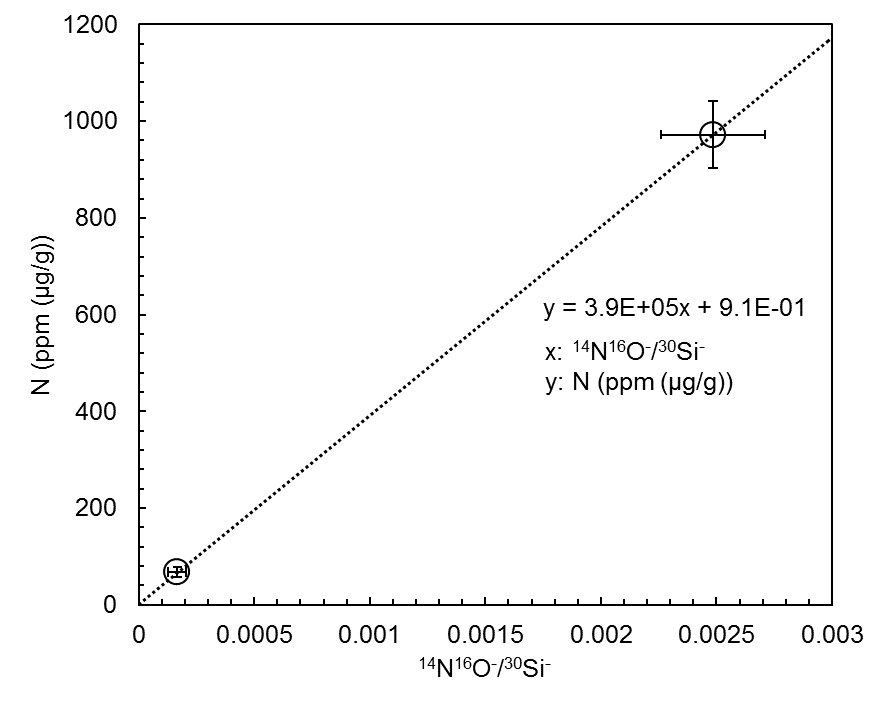


Supplementary Figure S2. Calibration line used for SIMS measurements of nitrogen concentration. Two ^14^N-implanted quartz glass samples were employed as standards.


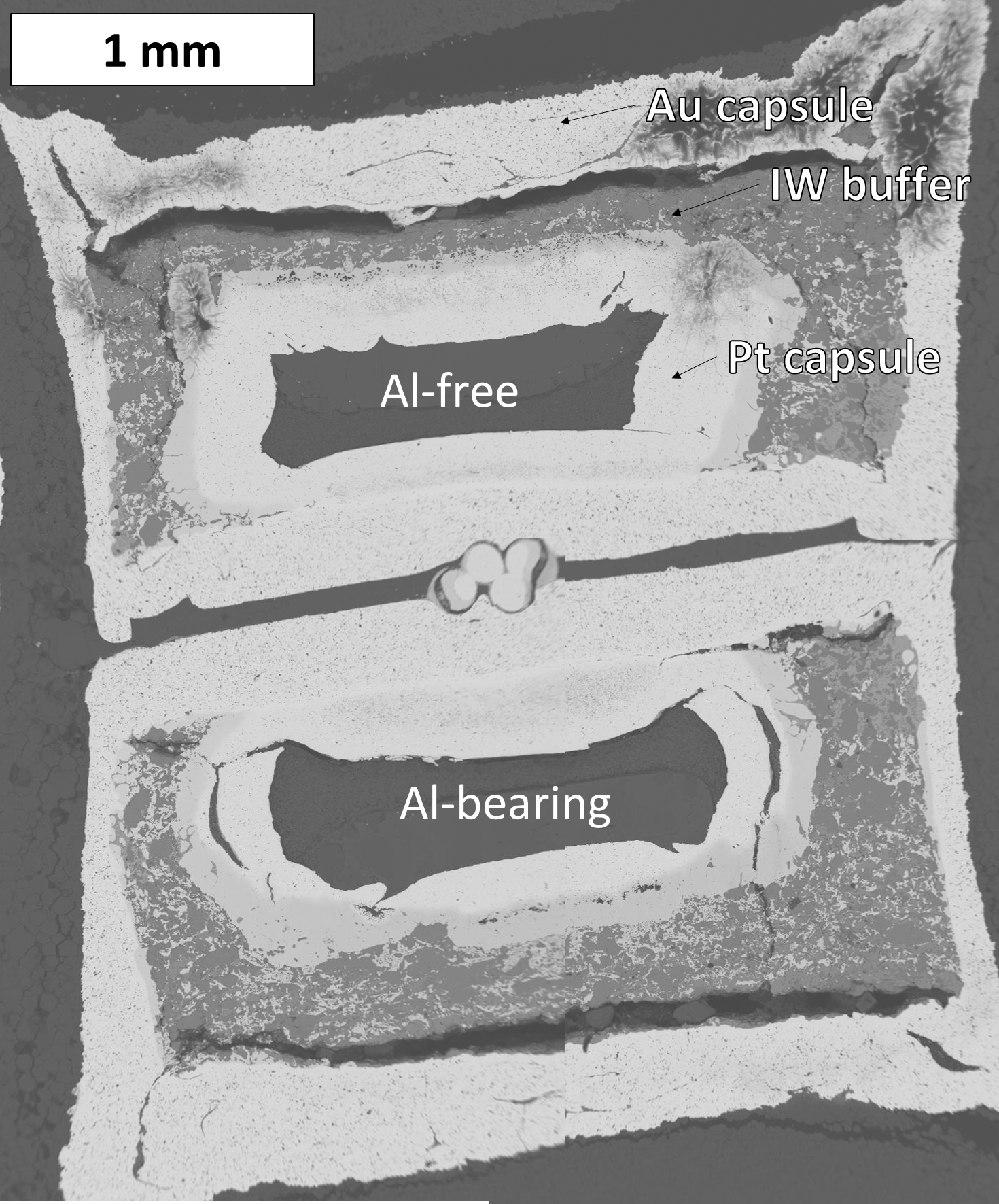


Supplementary Figure S3. BSE images of recovered sample from 28 GPa, 1500 °C (OT2258).

This figure was created by Ko Fukuyama using Microsoft Power Point (OFFICE 2016).


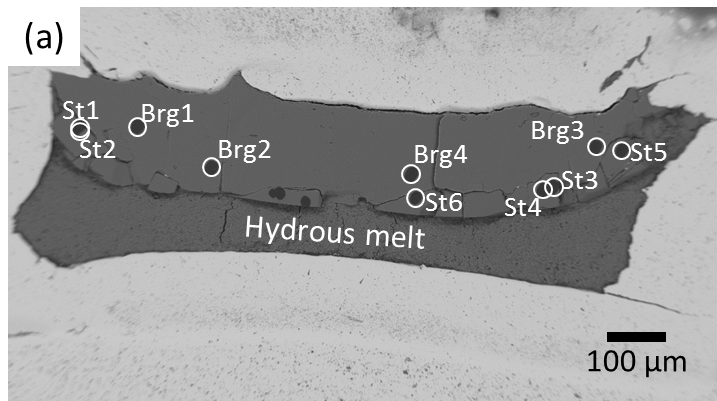


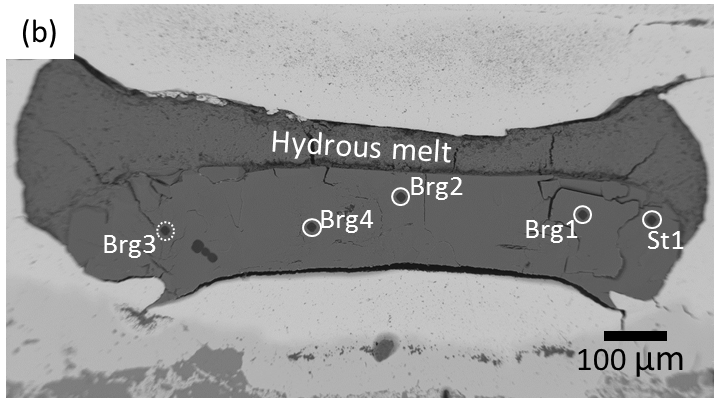


Supplementary Figure S4. BSE images of two samples obtained by FE-SEM-EDS in OT2258; Al-free system (a) and Al-bearing system (b). Circles responds to analysis points. St, stishovite; Brg, bridgmanite. Brg1-4 in the Al-bearing sample contained iron. Dotted circles were not discussed in this paper because these analysis points existed in cracks or grain boundaries.

This figure was created by Ko Fukuyama using Microsoft Power Point and Excel (OFFICE 2016).


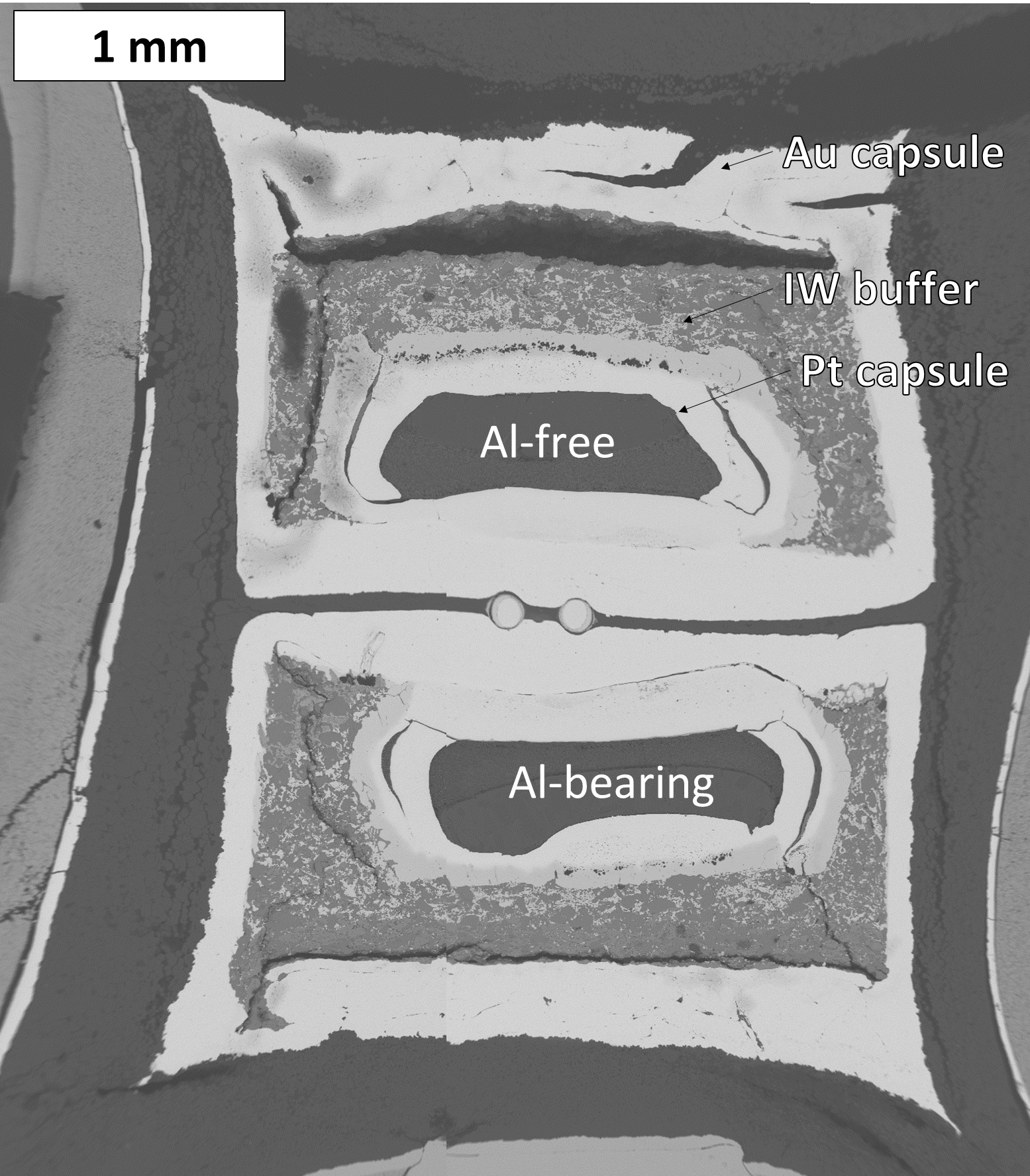


Supplementary Figure S5. BSE images of recovered sample from 28 GPa, 1620 °C (OT2293).

This figure was created by Ko Fukuyama using Microsoft Power Point (OFFICE 2016).


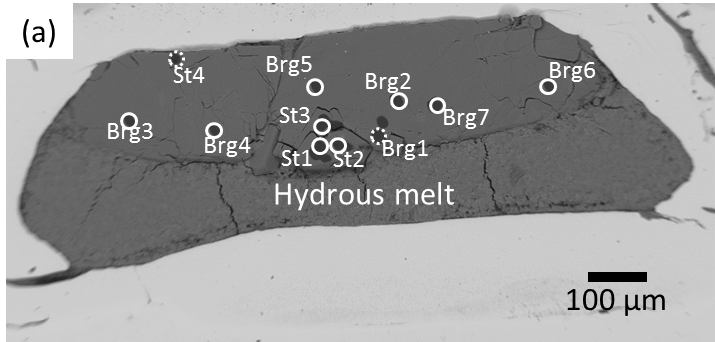


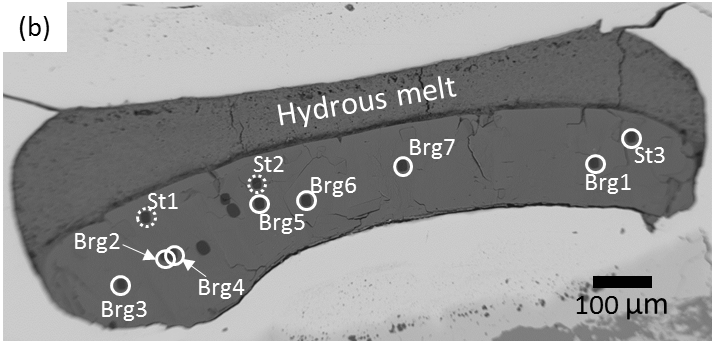


Supplementary Figure S6. BSE images of two samples obtained by FE-SEM-EDS in OT2293; Al-free system (a) and Al-bearing system (b). Circles responds to analysis points. St, stishovite; Brg, bridgmanite. Brg1-7 in Al-bearing sample contained iron. Dotted circles were not discussed in this paper because these analysis points existed in cracks or grain boundaries.

This figure was created by Ko Fukuyama using Microsoft Power Point (OFFICE 2016).


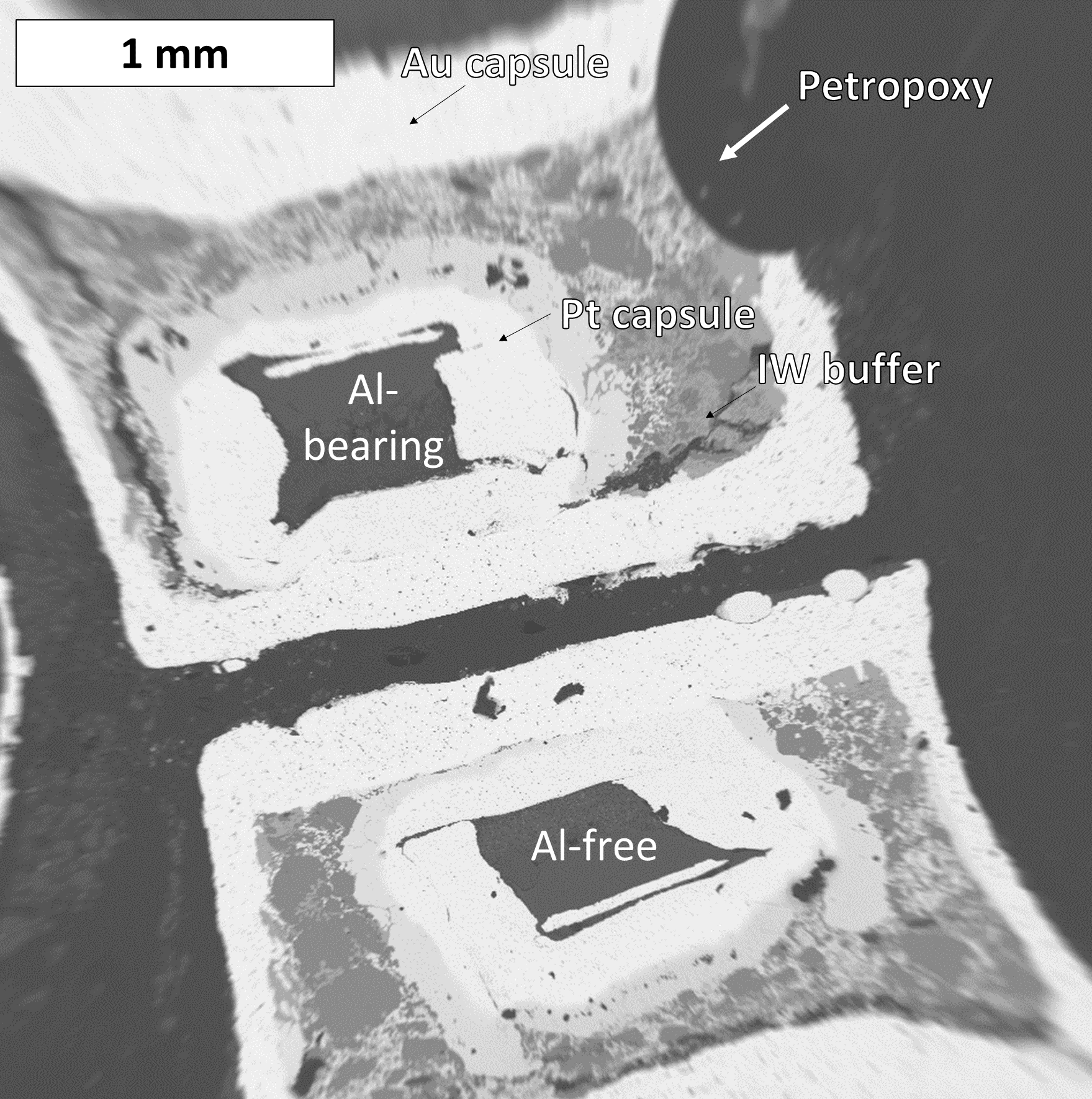


Supplementary Figure S7. BSE images of recovered sample from 28 GPa, 1700 °C (OS3083).

This figure was created by Ko Fukuyama using Microsoft Power Point (OFFICE 2016).


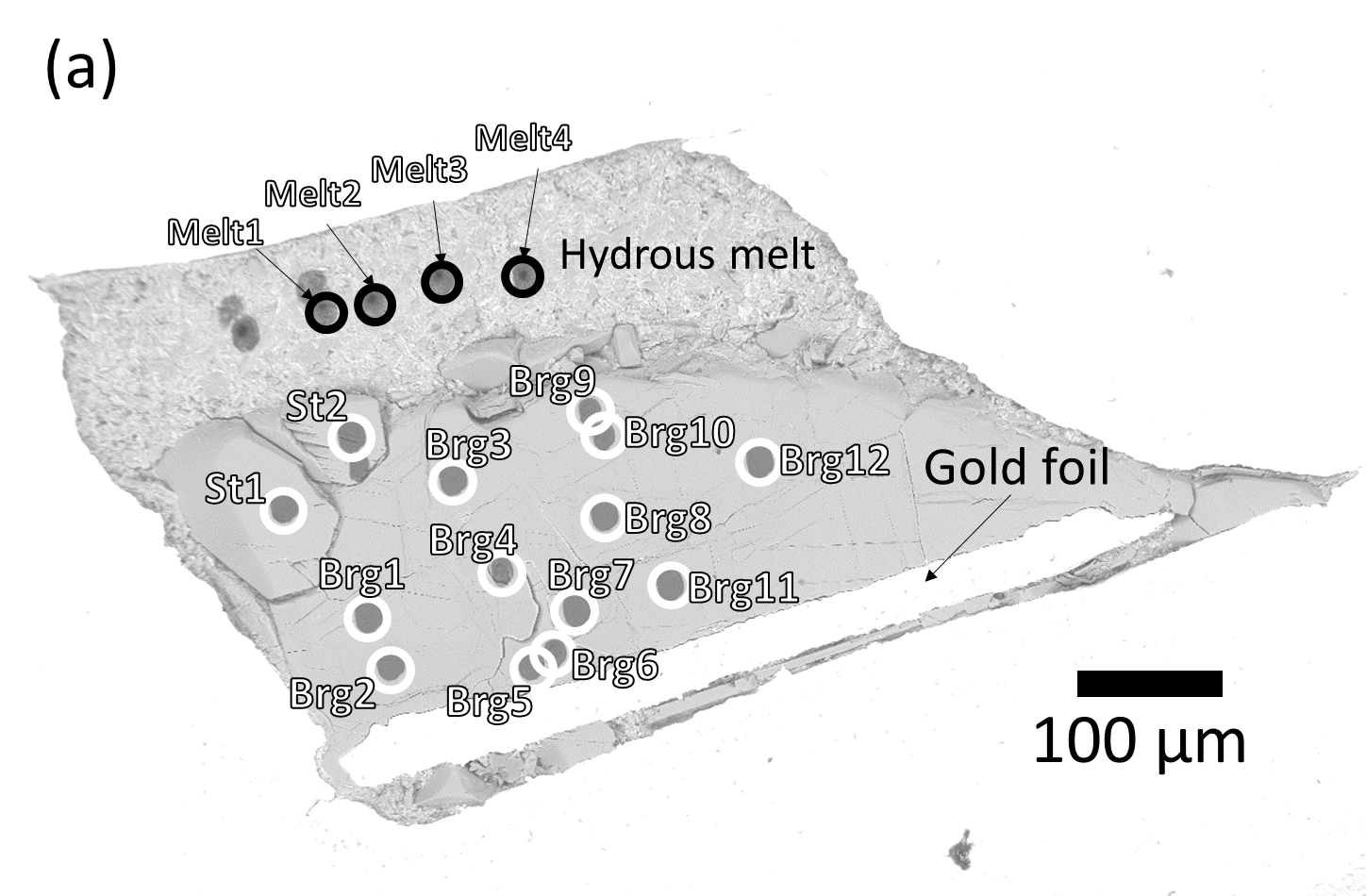


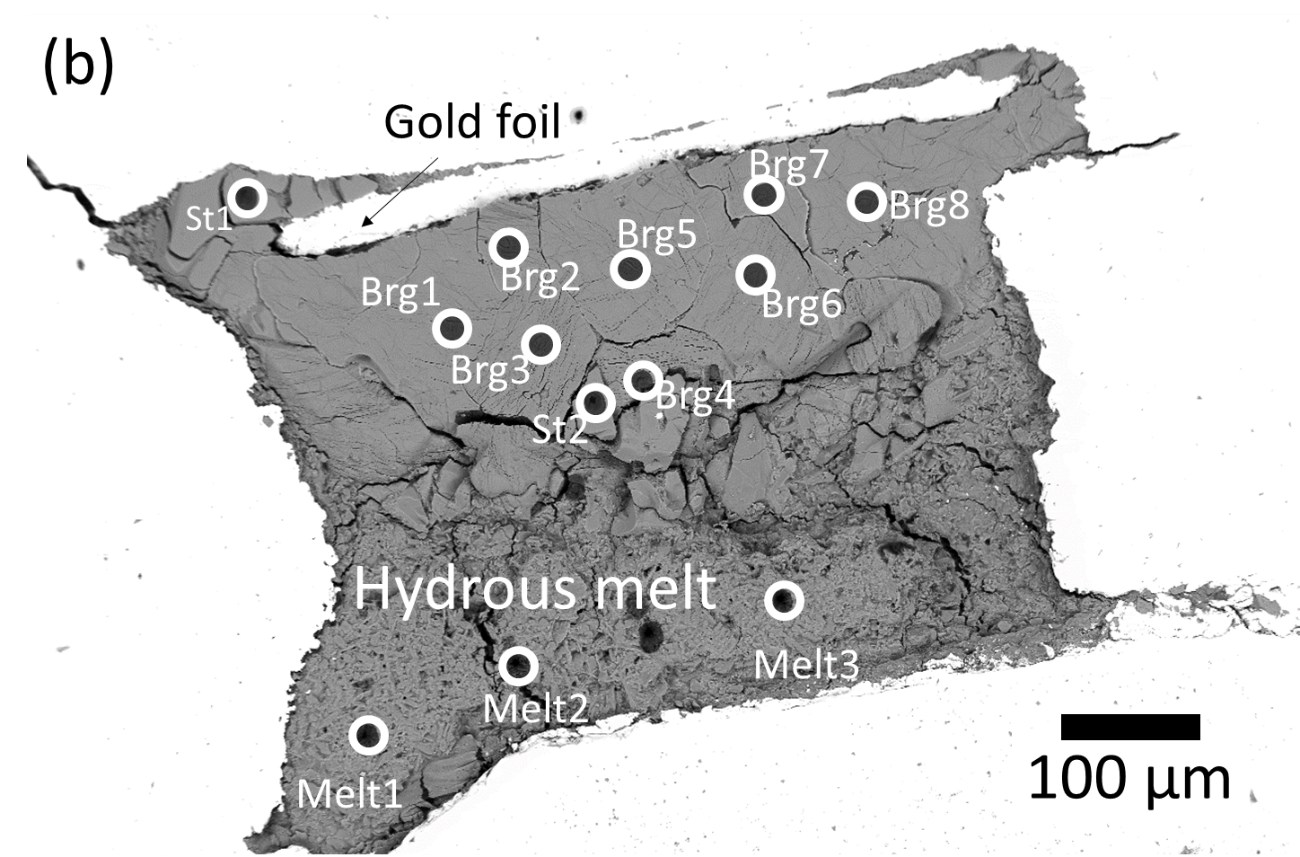


Supplementary Figure S8. BSE images of two samples obtained by FE-SEM-EDS in OS3083; Al-free system (a) and Al-bearing system (b). St, stishovite; Brg, bridgmanite.

This figure was created by Ko Fukuyama using Microsoft Power Point (OFFICE 2016).


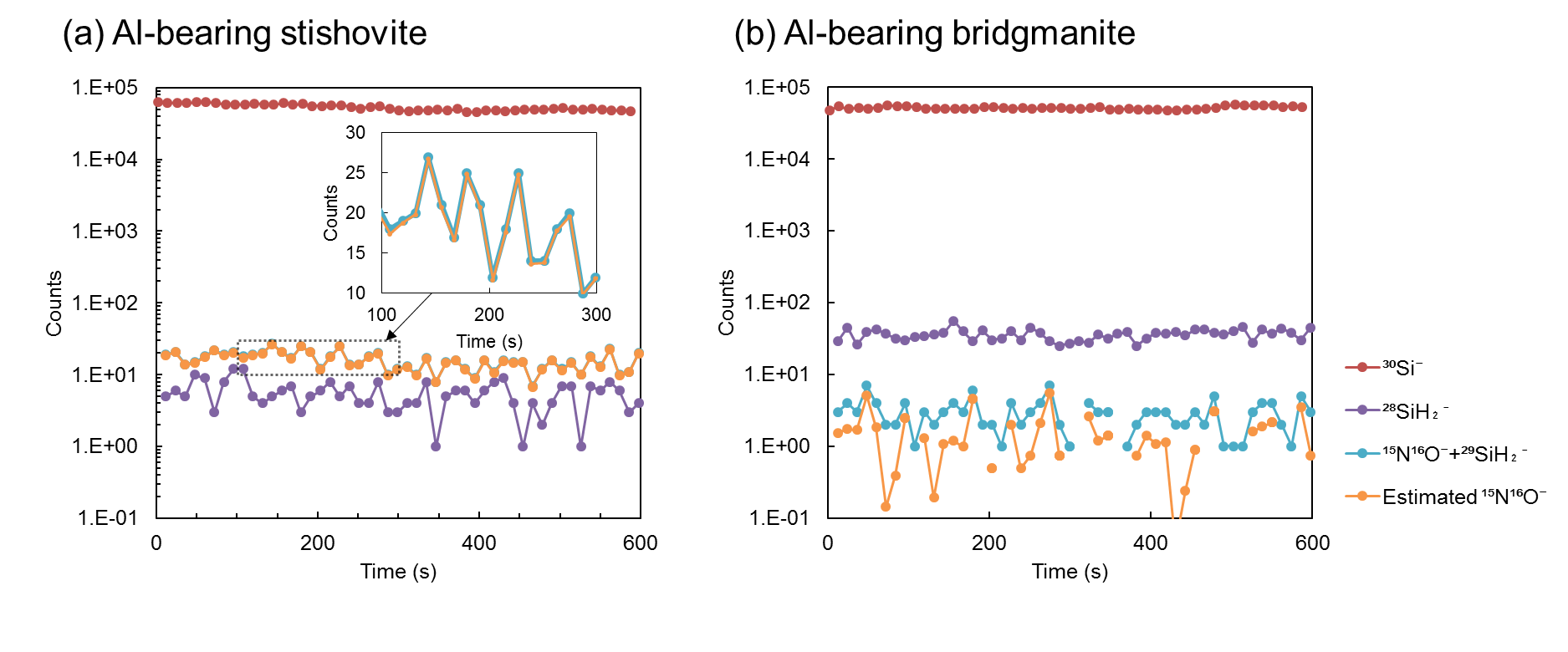


Supplementary Figure S9. Ion mass counts. (a): Al-bearing stishovite (OT2259_Al-St1) The inset shows enlarged trends for ion counts of ^15^N^16^O^-^ + ^29^SiH_2_^-^ and estimated ^15^N^16^O^-^. These two values coincide each other. (b): Al-bearing bridgmanite (OT2259_Al-Brg2).


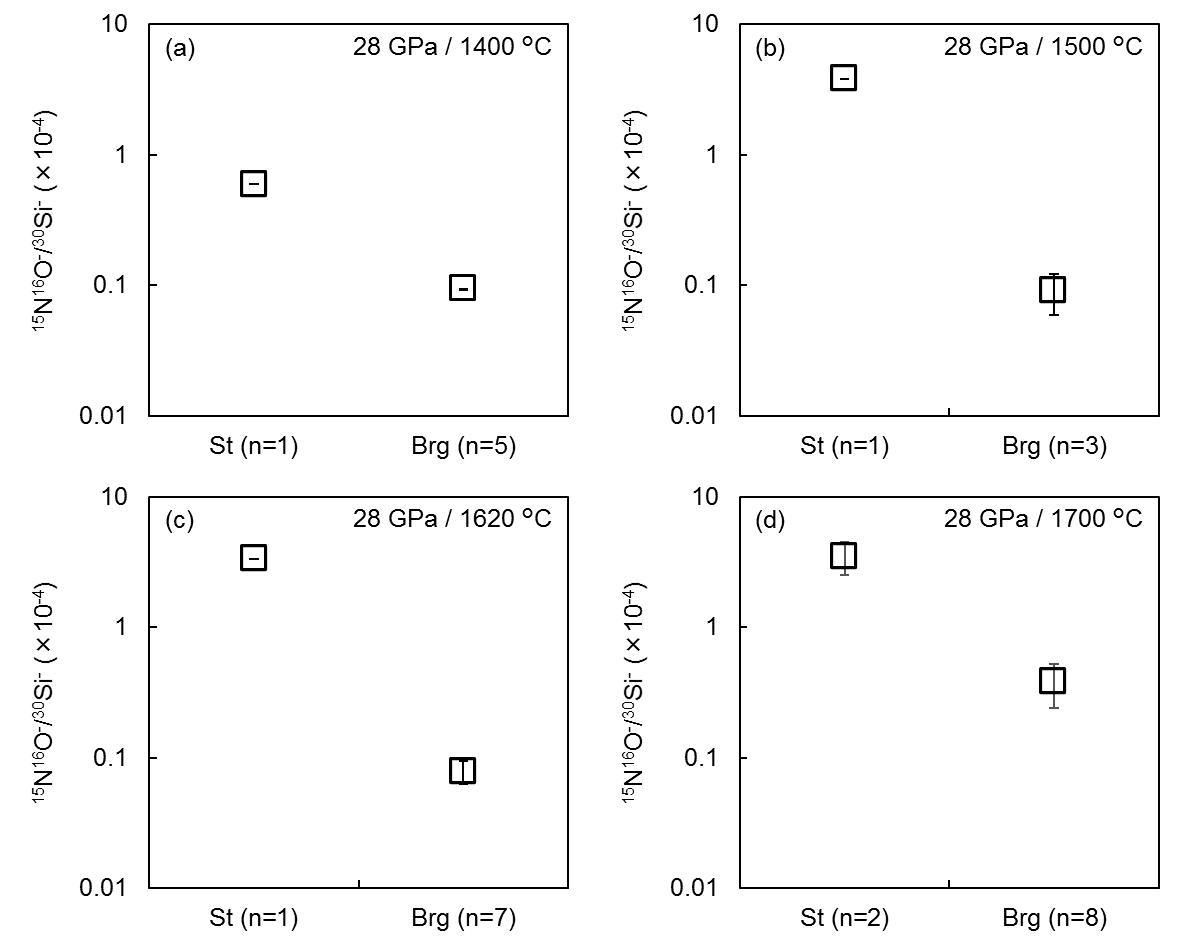


Supplementary Figure S10. Count ratio of ^15^N^16^O^-^ to ^30^Si^-^ in Al-bearing system (St = stishovite, Brg = bridgmanite). n corresponds to number of analysis points. a: Sample recovered from 28 GPa, 1400 °C (OT2259). b: Sample recovered from 28 GPa, 1500 °C (OT2258). c: Sample recovered from 28 GPa, 1620 °C (OT2293). d: Sample recovered from 28 GPa, 1700 °C (OS3083). St, stishovite; Brg, bridgmanite.


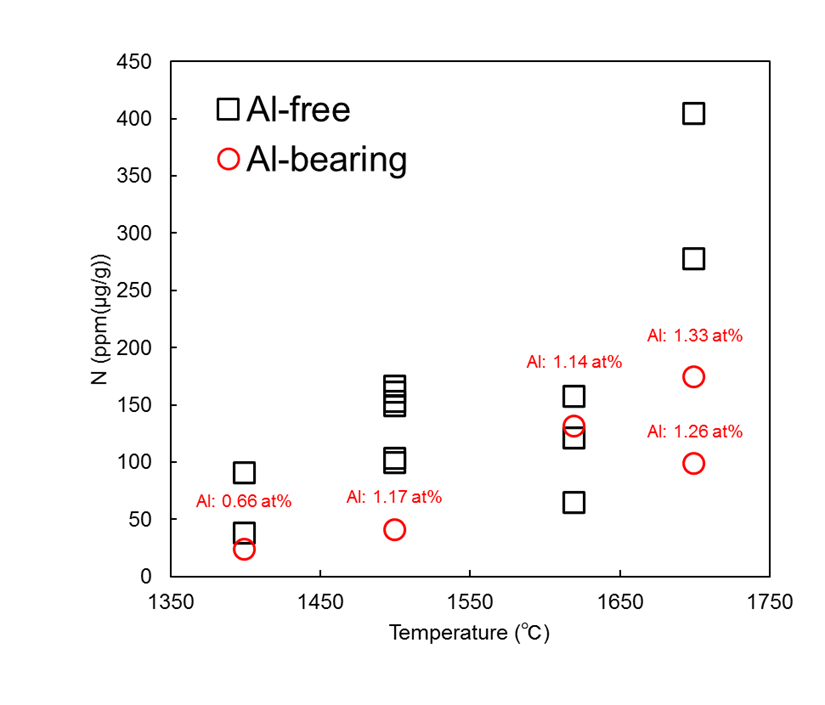
Supplementary Figure S11. Comparison of nitrogen solubilities between Stishovite (SiO_2_) and Al-bearing stishovite.

Supplementary Table S1. Chemical composition of bridgmanite, stishovite in recovered samples.

(a) OS3083 (28GPa, 1700 ºC)

Al-free system

|  | MgO | Al_2_O_3_ | SiO_2_ | FeO | total (wt.%) | N (ppm) |
| --- | --- | --- | --- | --- | --- | --- |
| St1 | 0.00 | 0.00 | 100 | 0.00 | 100 | 277 |
| St2 | 0.00 | 0.00 | 100 | 0.00 | 100 | 404 |
| Brg1 | 41.7 | 0.00 | 58.3 | 0.00 | 100 | ー |
| Brg2 | 41.5 | 0.00 | 58.5 | 0.00 | 100 | ー |
| Brg3 | 41.7 | 0.00 | 58.3 | 0.00 | 100 | ー |
| Brg4 | 42.1 | 0.00 | 57.9 | 0.00 | 100 | ー |
| Brg5 | 41.3 | 0.00 | 58.7 | 0.00 | 100 | ー |
| Brg6 | 41.0 | 0.00 | 59.0 | 0.00 | 100 | ー |
| Brg7 | 41.3 | 0.00 | 58.7 | 0.00 | 100 | ー |
| Brg8 | 42.7 | 0.00 | 57.3 | 0.00 | 100 | ー |
| Brg9 | 38.5 | 0.00 | 56.5 | 4.94 | 100 | ー |
| Brg10 | ー | ー | ー | ー | ー | ー |
| Brg11 | 40.5 | 0.00 | 59.5 | 0.00 | 100 | ー |
| Brg12 | 42.1 | 0.00 | 57.9 | 0.00 | 100 | ー |
| melt1 | 30.4 | 0.00 | 17.3 | 52.3 | 100 | ー |
| melt2 | 42.1 | 0.00 | 9.2 | 48.7 | 100 | ー |
| melt3 | 25.7 | 0.00 | 15.0 | 59.3 | 100 | ー |
| melt4 | 35.3 | 0.00 | 38.0 | 26.7 | 100 | ー |

*Some iron can be contaminated from Fe-FeO buffer in polishing recovered samples.

Al-bearing system

|  | MgO | Al_2_O_3_ | SiO_2_ | FeO | total (wt.%) | N (ppm) |
| --- | --- | --- | --- | --- | --- | --- |
| St1 | 0.00 | 3.71 | 96.3 | 0.00 | 100 | 174 |
| St2 | 0.00 | 3.22 | 96.8 | 0.00 | 100 | 98.1 |
| Brg1 | 42.7 | 3.63 | 53.6 | 0.00 | 100 | ー |
| Brg2 | 41.9 | 5.03 | 53.1 | 0.00 | 100 | ー |
| Brg3 | 44.4 | 1.96 | 53.7 | 0.00 | 100 | ー |
| Brg4 | 12.6 | 3.93 | 54.2 | 29.2 | 100 | ー |
| Brg5 | 43.9 | 1.57 | 54.6 | 0.00 | 100 | ー |
| Brg6 | 41.4 | 4.89 | 53.8 | 0.00 | 100 | ー |
| Brg7 | ー | ー | ー | ー | ー | ー |
| Brg8 | 43.6 | 2.19 | 54.3 | 0.00 | 100 | ー |
| melt1 | 69.0 | 10.9 | 16.0 | 4.18 | 100 | ー |
| melt2 | 95.1 | 4.86 | 0.00 | 0.00 | 100 | ー |
| melt3 | 67.5 | 7.72 | 9.15 | 15.6 | 100 | ー |

*Some iron can be contaminated from Fe-FeO buffer in polishing recovered samples.

(b) OT2293 (28GPa, 1620 ºC)

Al-free system

|  | MgO | Al_2_O_3_ | SiO_2_ | FeO | total (wt.%) | N (ppm) |
| --- | --- | --- | --- | --- | --- | --- |
| St1 | 0.00 | 0.00 | 100 | 0.00 | 100 | 157 |
| St2 | 0.00 | 0.00 | 100 | 0.00 | 100 | 120 |
| St3 | 0.00 | 0.00 | 100 | 0.00 | 100 | 63.7 |
| Brg2 | 44.2 | 0.00 | 55.9 | 0.00 | 100 | ー |
| Brg3 | 41.1 | 0.00 | 58.9 | 0.00 | 100 | ー |
| Brg4 | 41.0 | 0.00 | 59.0 | 0.00 | 100 | ー |
| Brg5 | 41.5 | 0.00 | 58.5 | 0.00 | 100 | ー |
| Brg6 | 44.1 | 0.00 | 55.9 | 0.00 | 100 | ー |
| Brg7 | 43.6 | 0.00 | 56.4 | 0.00 | 100 | ー |

Al-bearing system

|  | MgO | Al_2_O_3_ | SiO_2_ | FeO | total (wt.%) | N (ppm) |
| --- | --- | --- | --- | --- | --- | --- |
| St3 | 0.00 | 2.64 | 97.4 | 0.00 | 100 | 131 |
| Brg1 | 38.9 | 3.21 | 53.5 | 4.40 | 100 | ー |
| Brg2 | 44.0 | 2.10 | 53.9 | 0.00 | 100 | ー |
| Brg3 | 38.6 | 4.51 | 51.6 | 5.28 | 100 | ー |
| Brg4 | 42.4 | 2.90 | 53.4 | 1.32 | 100 | ー |
| Brg5 | 37.9 | 4.46 | 51.3 | 6.36 | 100 | ー |
| Brg6 | 42.7 | 1.57 | 54.0 | 1.66 | 100 | ー |
| Brg7 | 42.6 | 1.59 | 54.0 | 1.78 | 100 | ー |

(c) OT2258 (28GPa, 1500 ºC)

Al-free system

|  | MgO | Al_2_O_3_ | SiO_2_ | FeO | total (wt.%) | N (ppm) |
| --- | --- | --- | --- | --- | --- | --- |
| St1 | 0.78 | 0.00 | 99.2 | 0.00 | 100 | 166 |
| St2 | 0.78 | 0.00 | 99.2 | 0.00 | 100 | 160 |
| St3 | 0.00 | 0.00 | 100 | 0.00 | 100 | 99 |
| St4 | 0.00 | 0.00 | 100 | 0.00 | 100 | 102 |
| St5 | 0.00 | 0.00 | 100 | 0.00 | 100 | 153 |
| St6 | 0.00 | 0.00 | 100 | 0.00 | 100 | 149 |
| Brg1 | 44.0 | 0.00 | 56.0 | 0.00 | 100 | ー |
| Brg2 | 43.1 | 0.00 | 56.9 | 0.00 | 100 | ー |
| Brg3 | 43.4 | 0.00 | 56.6 | 0.00 | 100 | ー |
| Brg4 | 43.7 | 0.00 | 56.3 | 0.00 | 100 | ー |

Al-bearing system

|  | MgO | Al_2_O_3_ | SiO_2_ | FeO | total (wt.%) | N (ppm) |
| --- | --- | --- | --- | --- | --- | --- |
| St1 | 0.00 | 2.97 | 97.0 | 0.00 | 100 | 40.3 |
| Brg1 | 40.2 | 3.15 | 53.0 | 3.68 | 100 | ー |
| Brg2 | 37.3 | 4.63 | 51.2 | 6.84 | 100 | ー |
| Brg4 | 42.6 | 2.07 | 55.4 | 0.00 | 100 | ー |

(d) OT2259 (28 GPa, 1400 ºC)

Al-free system

|  | MgO | Al_2_O_3_ | SiO_2_ | FeO | total (wt.%) | N (ppm) |
| --- | --- | --- | --- | --- | --- | --- |
| St1 | 0.00 | 0.00 | 100 | 0.00 | 100 | 90.1 |
| St2 | 0.00 | 0.00 | 100 | 0.00 | 100 | 37.6 |
| Brg1 | 43.3 | 0.00 | 56.7 | 0.00 | 100 | ー |
| Brg2 | 44.1 | 0.00 | 55.9 | 0.00 | 100 | ー |
| Brg3 | 43.7 | 0.00 | 56.3 | 0.00 | 100 | ー |
| Brg4 | 43.6 | 0.00 | 56.5 | 0.00 | 100 | ー |
| Brg6 | 43.2 | 0.00 | 56.8 | 0.00 | 100 | ー |

Al-bearing system

|  | MgO | Al_2_O_3_ | SiO_2_ | FeO | total (wt.%) | N (ppm) |
| --- | --- | --- | --- | --- | --- | --- |
| St1 | 0.00 | 1.74 | 98.3 | 0.00 | 100 | 23.1 |
| Brg1 | 42.9 | 0.00 | 57.1 | 0.00 | 100 | ー |
| Brg2 | 37.8 | 5.44 | 50.0 | 6.75 | 100 | ー |
| Brg3 | 41.7 | 1.77 | 56.6 | 0.00 | 100 | ー |
| Brg4 | 38.4 | 4.83 | 50.8 | 5.98 | 100 | ー |
| Brg5 | 41.8 | 3.87 | 54.4 | 0.00 | 100 | ー |

**Supplementary References**

1. Katsura, T., Yoneda, A., Yamazaki, D., Yoshino, T. & Ito, E. Adiabatic temperature profile in the mantle. *Phys. Earth Planet. Inter.* **183**, 212–218 (2010).
2. Liu, X., Matsukage, K. N., Nishihara, Y., Suzuki, T. & Takahashi, E. Stability of the hydrous phases of Al-rich phase D and Al-rich phase H in deep subducted oceanic crust. *Am. Mineral.* **104**, 64–72 (2019).
3. Syracuse, E. M. *et al.* The global range of subduction zone thermal models. *Phys. Earth Planet. Inter.* **183**, 73–90 (2010).
